# Supplementary material for: A Global Screen for Assembly State Changes of the Mitotic Proteome by SEC-SWATH-MS
Source: Cell Syst. 2020 Feb 26;10(2):133–155.e6. doi: 10.1016/j.cels.2020.01.001 (PMC7042714; doi:10.1016/j.cels.2020.01.001)

Q9BZJ0 | CRNL1_HUMAN | CRNKL1 CRN CGI−201 MSTP021
Monomer MW [kDa]: 100.452 Monomer expected elution fraction: 40

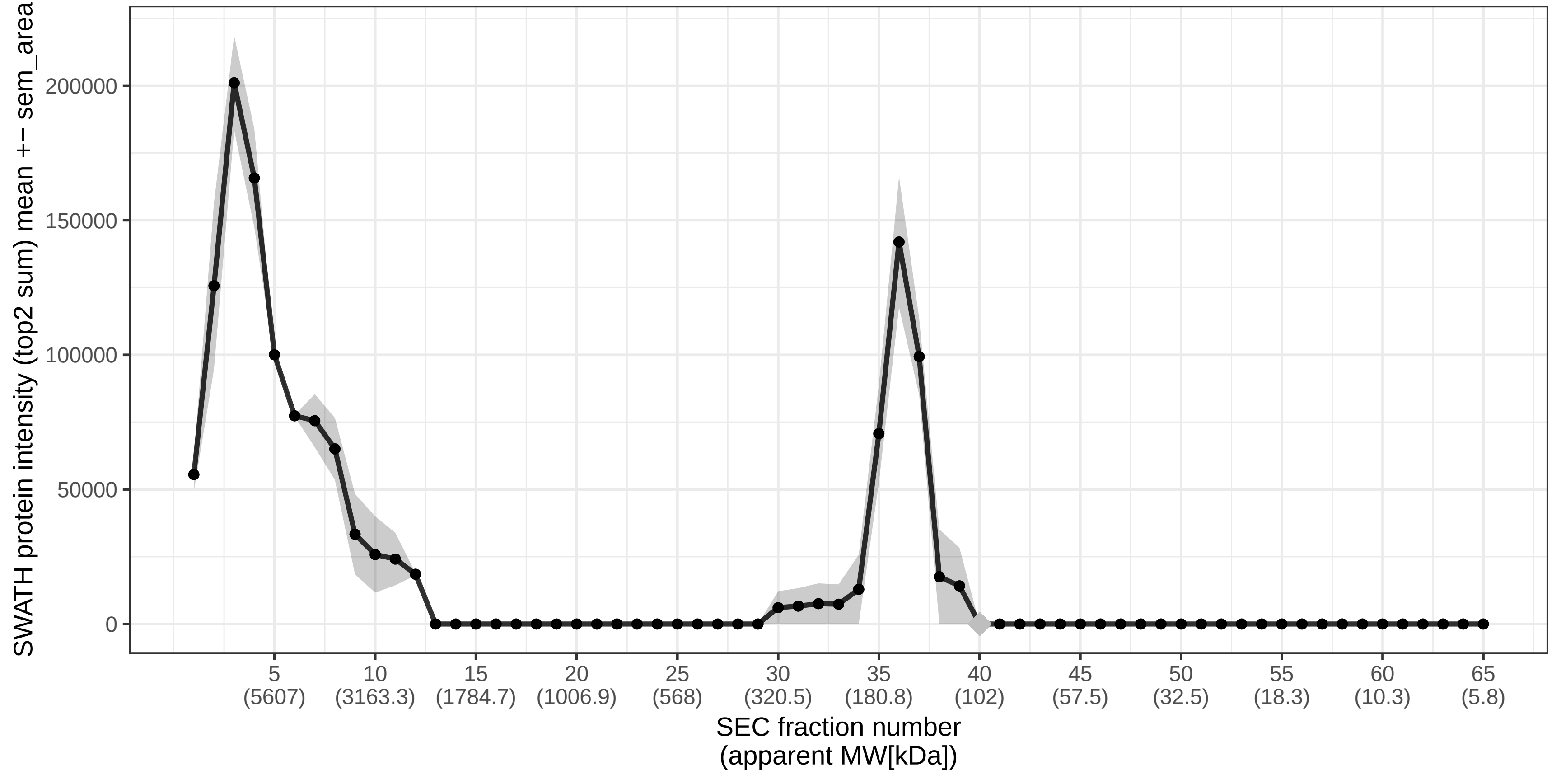

Supplement: Data S1. SEC-SWATH-MS Protein Chromatograms, Related to Figure 1 [file mmc6.zip › SECchrom_Q9BZJ0_CRNL1_HUMAN_CRNKL1_CRN_CGI-201_MSTP021.pdf]
